# Adjuvant chemoradiation in pancreatic cancer: impact of radiotherapy dose on survival

**DOI:** 10.1186/s12885-019-5790-2

**Published:** 2019-06-11

**Authors:** Alessio G. Morganti, Francesco Cellini, Milly Buwenge, Alessandra Arcelli, Sergio Alfieri, Felipe A. Calvo, Riccardo Casadei, Savino Cilla, Francesco Deodato, Giancarmine Di Gioia, Mariacristina Di Marco, Lorenzo Fuccio, Federica Bertini, Alessandra Guido, Joseph M. Herman, Gabriella Macchia, Bert W. Maidment, Robert C. Miller, Francesco Minni, Paolo Passoni, Chiara Valentini, Alessia Re, William F. Regine, Michele Reni, Massimo Falconi, Vincenzo Valentini, Gian Carlo Mattiucci

**Affiliations:** 1 Radiation Oncology Center, Department of Experimental, Diagnostic and Specialty Medicine-DIMES, University of Bologna, S. Orsola-Malpighi Hospital, via Giuseppe Massarenti 9, 40138 Bologna, Italy; 2 UOC Radioterapia Oncologica, Dipartimento di Diagnostica per immagini, Radioterapia Oncologica ed Ematologia, Istituto di Radiologia, Fondazione Policlinico A. Gemelli IRCCS, Università Cattolica Sacro Cuore, Roma, Italy; 3 Istituto di Clinica Chirurgica, Fondazione Policlinico A. Gemelli IRCCS - Università Cattolica Sacro Cuore, Roma, Italy; 4 Department of Oncology, Hospital General Universitario Gregorio Marañón, Complutense University, Madrid, Spain; 5 Department of Medical and Surgical Sciences – DIMEC, University of Bologna, Bologna, Italy; 6 Unit of Medical Physics, Fondazione Giovanni Paolo II, Campobasso, Italy; 7 Radiotherapy Unit, Fondazione Giovanni Paolo II, Campobasso, Italy; 8 Department of Experimental, Diagnostic, and Specialty Medicine - DIMES, Sant’Orsola-Malpighi Hospital, University of Bologna, Bologna, Italy; 9 Department of Radiation Oncology and Molecular Radiation Sciences, Johns Hopkins University School of Medicine, Baltimore, Maryland USA; 10 Department of Radiation Oncology, University of Virginia, Charlottesville, Virginia USA; 11 Department of Radiation Oncology, Mayo Clinic, Rochester, MN USA; 12 IRCCS, Ospedale S. Raffaele, Milan, Italy; 13 Department of Radiotherapy and Radiation Oncology, Faculty of Medicine and University Hospital Carl Gustav Carus, Technische Universität Dresden, Dresden, Germany; 14 Department of Radiation Oncology, University of Maryland Medical Center, Baltimore, MD USA; 15 Pancreatic Surgery, Pancreas Translational & Clinical Research Center, San Raffaele Hospital, University “Vita e Salute”, Milan, Italy

**Keywords:** Pancreatic neoplasm, Radiotherapy, Adjuvant, Dose effect

## Abstract

**Background:**

To evaluate the impact of radiation dose on overall survival (OS) in patients treated with adjuvant chemoradiation (CRT) for pancreatic ductal adenocarcinoma (PDAC).

**Methods:**

A multicenter retrospective analysis on 514 patients with PDAC (T1–4; N0–1; M0) treated with surgical resection with macroscopically negative margins (R0–1) followed by adjuvant CRT was performed. Patients were stratified into 4 groups based on radiotherapy doses (group 1: < 45 Gy, group 2: ≥ 45 and < 50 Gy, group 3: ≥ 50 and < 55 Gy, group 4: ≥ 55 Gy). Adjuvant chemotherapy was prescribed to 141 patients. Survival functions were plotted using the Kaplan-Meier method and compared through the log-rank test.

**Results:**

Median follow-up was 35 months (range: 3–120 months). At univariate analysis, a worse OS was recorded in patients with higher preoperative Ca 19.9 levels (≥ 90 U/ml; *p* < 0.001), higher tumor grade (G3–4, *p* = 0.004), R1 resection (*p* = 0.004), higher pT stage (pT3–4, *p* = 0.002) and positive nodes (*p* < 0.001). Furthermore, patients receiving increasing doses of CRT showed a significantly improved OS. In groups 1, 2, 3, and 4, median OS was 13.0 months, 21.0 months, 22.0 months, and 28.0 months, respectively (*p* = 0.004). The significant impact of higher dose was confirmed by multivariate analysis.

**Conclusions:**

Increasing doses of CRT seems to favorably impact on OS in adjuvant setting. The conflicting results of randomized trials on adjuvant CRT in PDAC could be due to < 45 Gy dose generally used.

## Background

Pancreatic carcinoma is projected to become the second leading cause of cancer mortality by 2030 with a 5-year overall survival (OS) rate around 7% [1]. At diagnosis, around 20% of pancreatic cancer patients present with a resectable tumor, 30% with a locally advanced tumor and 50% with metastatic disease [2]. Radical surgery with tumor-free margins is the only treatment with the potential to achieve long-term survival [3].

Nevertheless, both local or distant relapses commonly affect patients’ survival [4]. Therefore, local and systemic adjuvant treatments have been proposed to improve OS. Many clinical trials evaluated the efficacy of adjuvant chemo-radiotherapy (CRT) and chemotherapy (CT).

The efficacy of CT has been demonstrated [5–7] but the potential impact of CRT in the adjuvant setting remains controversial. In fact, an improved OS after postoperative CRT was described in several reports including: randomized trials as the Gastrointestinal Tumor Study group (GITSG) [8, 9] and European Organization for Research and Treatment of Cancer (EORTC) [10, 11] trials, single center analyses [12–14], meta-analyses [15] or pooled analyses [16, 17], and tumor registry studies [18–22].

On the other hand, the European Study Group for Pancreatic Cancer-1 (ESPAC-1) trial reported negative results after adjuvant CRT [5, 23, 24]. Characteristic of this study was the prescription of a relatively low dose of radiotherapy (RT) (40 Gy with split course regimen) similar to the previous GITSG [8, 9] and EORTC [10, 11] trials. It could be hypothesized that this dose was ineffective in improving local control (LC) of the disease and ultimately OS as suggested by some studies [25]. In fact, in the GITSG study, the incidence of local recurrence was 33% in patients who underwent surgery alone and 49% in patients undergoing adjuvant CRT and CT [9]. However, only few studies evaluated the impact of postoperative CRT dose on clinical outcome [25, 26].

Our previous pooled analysis confirmed the positive impact of adjuvant CRT on OS [27]. Therefore, on the basis of the above considerations, a secondary analysis of that study was performed in order to assess the impact of CRT dose on clinical outcome in terms of OS. The purpose of this paper is to report the results of this secondary analysis.

## Methods

### Study design and participants

Clinical data from 7 different institutions (Baltimore [2 institutions], Rochester, Madrid, Rome, Campobasso, Milan) were retrospectively pooled for this analysis on individual patient basis. Treatment was delivered between 1995 and 2008.

**Fig. 1:**
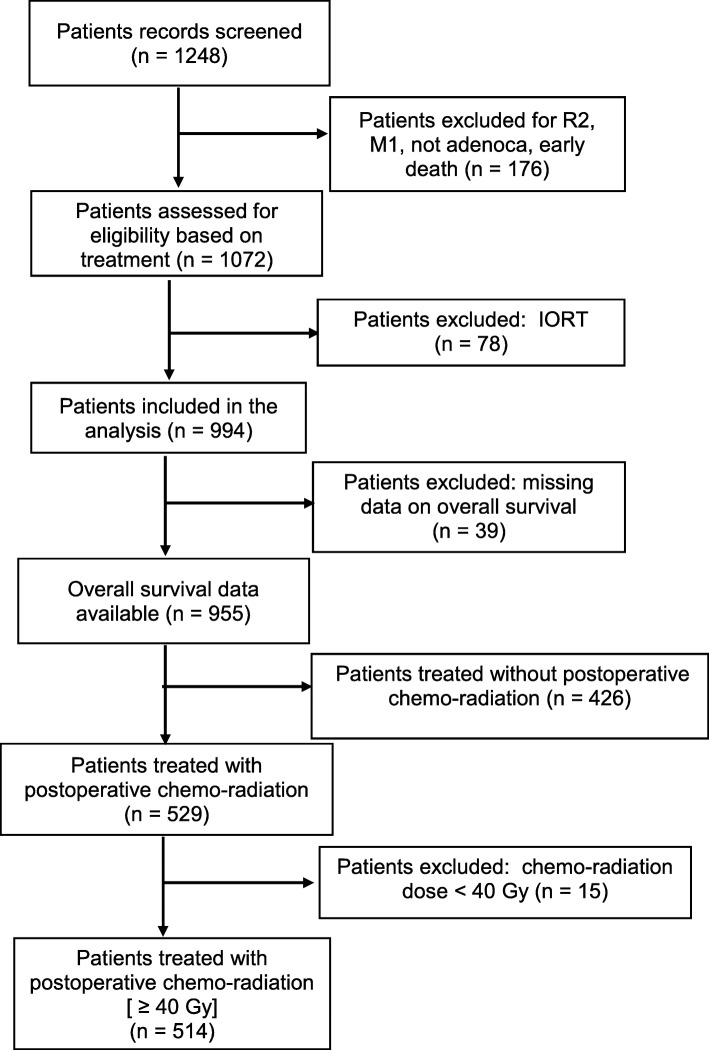
Flow-chart of patients’ selection

The following exclusion criteria were used: metastatic disease (M1), diagnoses different from pancreatic ductal adenocarcinoma (PDAC), neoadjuvant treatment and/or intraoperative radiation therapy, postoperative CRT dose < 40 Gy, death within 60 days of surgery, and missing data on pathological tumor (pT) stage and/or nodal status. By excluding patients with missing data on survival and/or not eligible, 955 patients remained in our reference database. In this analysis, only 514 patients receiving postoperative CRT (Fig. 1) were included.

The following variables were analyzed: gender, age, tumor location (head, body, tail), tumor grade (I-IV), tumor diameter (mm), surgical procedure (pancreaticoduodenectomy, distal, total pancreatectomy), pT and nodal stage and presence of microscopic residual disease. Patients were stratified into 4 classes based on RT doses (< 45 Gy, ≥ 45 and < 50 Gy, ≥ 50 and < 55 Gy, ≥ 55 Gy).

The first cut-off of 45 Gy was applied to discriminate patients treated with dosage similar to the most important randomized trials [5, 8, 10]. The second cut-off of 50 Gy represents the dosage recommended by international guidelines [28]. The third cut-off of 55 Gy was selected because a substantial number of patients received higher doses based on the personal experience of radiation oncologists, their technological equipment, and due to the higher risk of local recurrence.

### Adjuvant CRT

The details of adjuvant RT have been described in detail elsewhere [13, 14, 17, 27, 29–31]. In brief, adjuvant external-beam RT was delivered with linear accelerators on tumor bed and regional nodes using multiple-field techniques. CRT dose ranged between 40.0 and 61.2 Gy (median: 50.4 Gy). Most patients underwent 3D-conformal therapy while no patient received treatment based on Intensity Modulated RT or Volumetric Arc Therapy. All patients received a continuous course of CRT without a planned break. The dose was prescribed according to the guidelines of International Commission on Radiation Units Measurements Report 50. In most centers concurrent CT was based on 5-fluorouracil or capecitabine and adjuvant CT was mainly based on gemcitabine.

### End-points

The primary end-point was OS calculated from the date of diagnosis. Secondary aim was to investigate factors associated with OS.

### Statistical analysis

Descriptive statistics included frequencies and percentages for categorical variables and means plus standard deviations or medians and range for continuous variables. Chi-square Pearson analysis was used to determine any statistical significance between the distributions of categorical variables while Kruskal-Wallis test was undertaken to determine any statistical significance for continuous variable. Survival functions were plotted using the Kaplan-Meier method [32] and compared through the log-rank test [33] to investigate differences in OS between groups defined based on clinical and pathological factors. Clinical and pathological parameters associated with significant differences in OS at the univariate analysis were entered into a multivariable Cox model using a forward stepwise [Wald] strategy [34] (p removal ≥0.10; *p* addition < 0.05) based on likelihood ratio test in order to obtain a final model including only the subset of variables significant in predicting OS. All tests were two-sided and a *p* value < 0.05 was considered statistically significant. Statistical analysis was performed with IBM SPSS (IBM SPSS Statistics for Windows, Inc., Version 20.0; IBM Corp, Armonk, NY, USA).

## Results

Median follow-up time was 35 months (range: 3–120 months). The median age for the entire cohort of 514 patients was 63 years (range: 29–85 years). No differences between patients receiving different RT dose (< 45 Gy, ≥ 45 and < 50 Gy, ≥ 50 and < 55 Gy, ≥ 55 Gy) were observed in terms of median age, mean tumor diameter and tumor site while type of resection (*p* < 0.001), grading (*p* < 0.001), rate of R1 resection (*p* = 0.032), tumor stage (*p* = 0.006), incidence of lymph nodes involvement (*p* = 0.001), and adjuvant CT treatment (*p* < 0.001) were different between the groups. In particular, the cohort of patients who received a dose ≥ 55 Gy differed significantly from the other groups both for more unfavourable prognostic characteristics (higher percentage of patients with positive margins, with tumor diameter ≥ 30 mm, with pT4 and pN+ stage) and for an increased use of adjuvant CT (Table 1). Concurrent CT was based on 5-FU regimen in 71.6% of patients, while 28.4% of patients were treated with different regimens: gemcitabine (14.4%), capecitabine (9.5%), gemcitabine + 5-FU (3.1%) and tegafur (1.4%). All patients who underwent adjuvant CT were treated with gemcitabine.

**Table 1:** Patients characteristics: number [No] and percentages [%] of patients

Variable	Value	No of patients [%]	No of patients [%] treated with < 45 Gy	No of patients [%] treated with ≥ 45 and < 50 Gy	No of patients [%] treated with ≥ 50 and < 55 Gy	No of patients [%] treated with ≥ 55 Gy	*p* value
Age	Median (range)	63.0 (29–85)	64.4 (38–81)	64.0 (41–84)	63.9 (29–86)	62.0 (35–78)	.479
Tumor diameter (cm)	Mean (SD)		2.82 ± 1.51	2.95 ± 1.48	3.12 ± 1.42	3.00 ± 1.31	.190
Gender	Male	239 [46.5]	11 [42.3]	31 [43.1]	156 [47.0]	41 [48.8]	.410
	Female	275 [53.5]	15 [57.7]	41 [56.9]	176 [53.0]	43 [51.2]	
Ca 19.9 (units/mL)	< 90	114 [22.1]	5 [19.2]	21 [29.2]	67 [20.2]	21 [25.0].	.076
	≥ 90	137 [26.7]	4 [15.4]	16 [22.2]	83 [25.0]	34 [40.5]	
	Unknown	263 [51.2]	17 [65.4]	35 [48.6]	182 [54.8]	29 [34.5]	
Tumor site	Head	432 [84.0]	22 [84.6]	66 [91.6]	272 [81.9]	72 [85.7]	.408
	Body	26 [5.1]	2 [7.7]	1 [1.4]	17 [5.1]	6 [7.1]	
	Tail	36 [7.0]	2 [7.7]	2 [2.8]	27 [8.2]	5 [6.0]	
	Unknown	20 [3.9]	0 [0.0]	3 [4.2]	16 [4.8]	1 [1.2]	
Type of resection	DCP	315 [61.2]	14 [53.8]	41 [56.9]	190 [57.2]	70 [83.3]	<.001
	DP	61 [11.9]	3 [11.6]	4 [5.6]	43 [13.0]	11 [13.1]	
	TP	21 [4.1]	4 [15.4]	4 [5.6]	12 [3.6]	1 [1.2]	
	PPP	117 [22.8]	5 [19.2]	23 [31.9]	87 [26.2]	2 [2.4]	
Grading	1	34 [6.6]	1 [3.8]	5 [7.0]	12 [3.6]	16 [19.0]	<.001
	2	119 [23.2]	5 [19.2]	18 [25.0]	65 [19.6]	31 [36.9]	
	3	250 [48.6]	13 [50.0]	38 [52.7]	171 [51.5]	28 [33.3]	
	4	45 [8.8]	1 [3.8]	6 [8.3]	35 [10.5]	3 [3.6]	
	Unknown	66 [12.8]	6 [23.2]	5 [7.0]	49 [14.8]	6 [7.2]	
Margins status	R0	132 [25.7]	8 [30.8]	14 [19.4]	70 [21.1]	40 [47.6]	.032
	R1	61 [11.9]	3 [11.5]	4 [5.6]	22 [6.6]	32 [38.1]	
	Unknown	321 [62.4]	15 [57.7]	54 [75.0]	240 [72.3]	12 [14.3]	
Tumor diameter	< 30 mm	68 [13.2]	5 [19.2]	9 [12.5]	17 [5.1]	37 [44.0]	.206
	≥ 30 mm	61 [11.9]	2 [7.7]	4 [5.6]	24 [7.2]	31 [36.9]	
	Unknown	385 [74.9]	19 [73.1]	59 [81.9]	291 [87.7]	16 [19.1]	
pT-stage	1	33 [6.4]	1 [3.8]	5 [6.9]	26 [7.9]	1 [1.2]	.006
	2	107 [20.8]	4 [15.4]	15 [20.8]	81 [24.4]	7 [8.3]	
	3	341 [66.3]	21 [80.8]	48 [66.7]	206 [62.0]	66 [78.6]	
	4	33 [6.4]	0 [0.0]	4 [5.6]	19 [5.7]	10 [11.9]	
pN-stage	N0	205 [39.9]	9 [34.6]	28 [38.9]	150 [45.2]	18 [21.4]	.001
	N+	309 [60.1]	17 [65.4]	44 [61.1]	182 [54.8]	66 [78.6]	
Adjuvant CT	No	308 [59.9]	19 [73.1]	48 [66.7]	227 [68.4]	14 [16.7]	<.001
	Yes	141 [27.5]	3 [11.5]	18 [25.0]	54 [16.2]	66 [78.6]	
	Unknown	65 [12.6]	4 [15.4]	6 [8.3]	51 [15.4]	4 [4.7]	

*DCP* duodenocephalopancreasectomy, *DP* distal pancreatectomy, *TP* total pancreatectomy, *PPP* pylorus preserving pancreatectomy, *CT* chemotherapy

### Overall survival

**Fig. 2:**
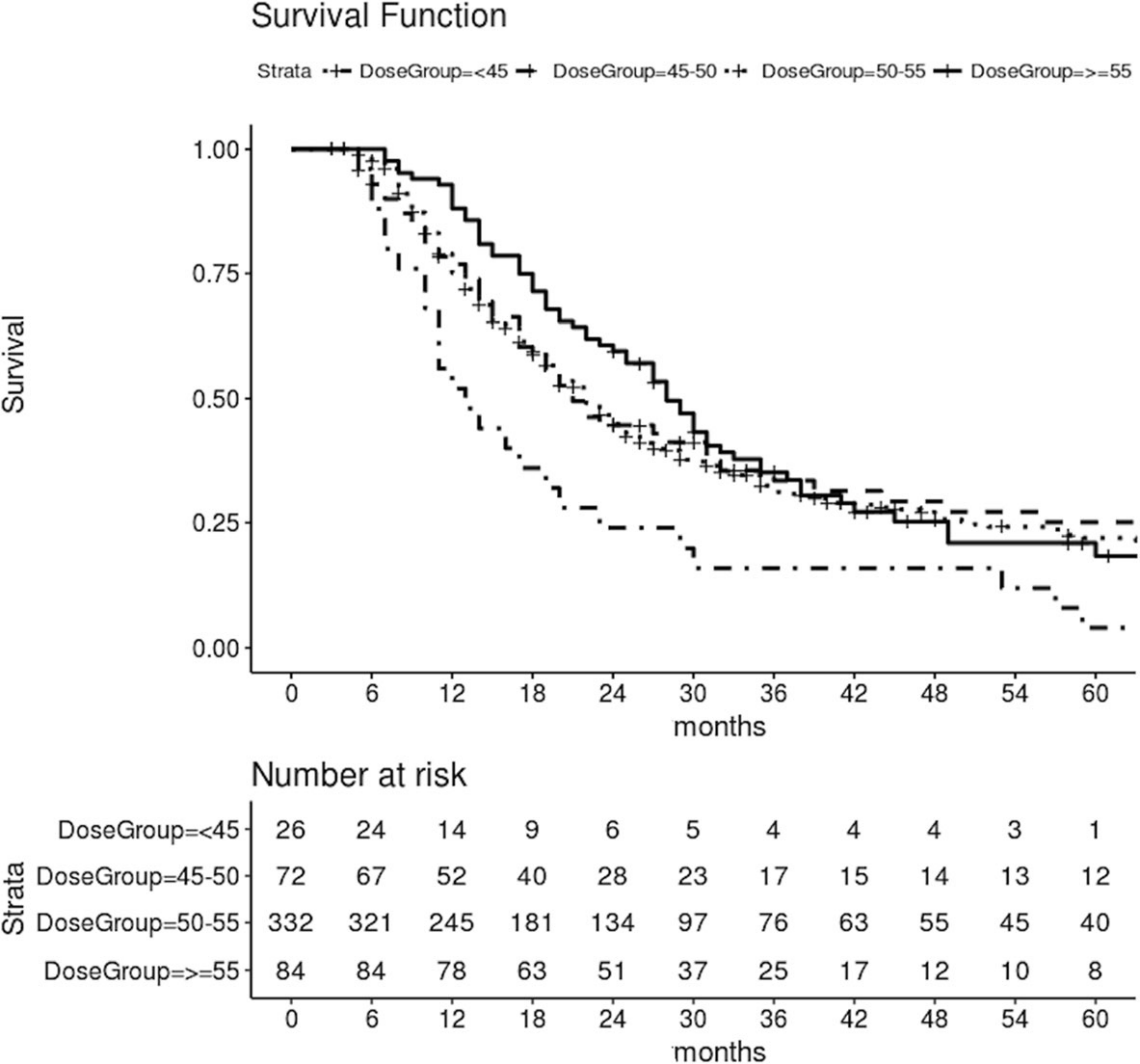
Overall survival of patients who received a chemo-radiation dose < 45 Gy, or ≥ 45 Gy or < 50 Gy, or ≥ 50 Gy and < 55 Gy, or ≥ 55 Gy

Patients receiving increasing doses of CRT showed a significantly improved OS (Fig. 2). Median OS was 13.0 months (95% CI, 8.1–17.8 months) with < 45 Gy CRT dose, versus 21.0 months (95% CI, 16.6–25.3 months) with ≥ 45 and < 50 Gy CRT dose, versus 22.0 months (95% CI, 19.6–24.3 months) with ≥ 50 and < 55 Gy CRT dose, versus 28.0 months (95% CI, 24.1–31.8 months) with ≥ 55 Gy CRT dose (*p* = 0.004).

A better prognosis was recorded in patients with preoperative Ca 19.9 level <  90 U/ml, lower tumor grade, R0 resection, lower pT stage, negative lymph nodes and who received adjuvant CT. Table 2 shows survival differences at univariate analysis based on clinical, pathological and treatment details in the whole population.

**Table 2:** Univariate analysis including 2-, 5-year, and median overall survival (OS) time

Variable	Value	No. of patients	2-year OS (%)	5-year OS (%)	Median OS (months)	*P* value
Gender	Male	239	50.0	23.8	25	.084
	Female	275	43.1	18.0	21	
Ca 19.9 (units/mL)	< 90	114	62.6	31.5	32	.000
	≥ 90	137	39.8	18.4	21	
Tumor Site	Head	432	47.0	20.9	23	.909
	Body	26	40.4	11.8	21	
	Tail	36	44.3	15.8	22	
Type of resection	DCP	315	48.1	22.9	23	.090
	DP	61	45.1	17.4	22	
	TP	21	20.8	10.4	16	
	PPP	117	47.0	18.0	22	
Grading	1	34	61.8	39.2	29	.004
	2	119	51.9	19.3	25	
	3	250	43.7	19.3	20	
	4	45	35.1	19.3	15	
Margins status	R0	132	59.7	25.2	29	.004
	R1	61	36.8	10.8	20	
Tumor diameter (mm)	< 30	68	57.7	15.5	28	.529
	≥ 30	61	48.4	20.3	24	
pT-stage	1	33	70.3	41.9	45	.002
	2	107	54.4	26.3	28	
	3	341	42.7	17.5	21	
	4	33	33.3	14.5	18	
pN-stage	N0	205	56.6	30.3	30	.000
	N+	309	39.6	14.4	20	
Adjuvant CT	No	308	43.1	21.1	20	.004
	Yes	141	53.7	18.8	26	
RT dose (Gy)	< 45	26	24.0	4.0	13	.004
	≥ 45 and < 50	72	44.6	25.1	21	
	≥ 50 and < 55	332	44.9	22.0	22	
	≥ 55	84	59.5	18.4	28	

*CT* chemotherapy, *DCP* duodenocephalopancreatectomy, *DP* distal pancreatectomy, *OS* overall survival, *PPP* pylorus preserving pancreatectomy, *RT* radiotherapy, *TP* total pancreatectomy

To assess the impact of the different CRT dose on OS in patients’ subsets, patients were stratified in subgroups based on potentially predictive variables. The positive impact of higher doses was confirmed with statistical significance in female patients, both lower and higher Ca 19.9 subgroups, in patients with PDAC of the pancreatic head, in patients treated with duodenocephalopancreatectomy and total pancreatectomy, in grade 1 and 3 PDAC, in both patients’ groups with negative and positive resection margins, in tumors < 30 mm diameter, in patients with pT3 and pN0 cancer, and receiving adjuvant CT (Table 3).

**Table 3:** Sub analysis of all predictor values of 2-, 5-year, and median overall survival (OS). Data are stratified for postoperative chemo-radiation dose (< 45 Gy, > 45 and 50 Gy, ≥ 50 and < 55 Gy, ≥ 55 Gy)

Variable	Value	OS								Median OS (months)				*p* value
		2-y (%)				5-y (%)								
RT dose (Gy)		< 45	≥ 45 and < 50	≥ 50 and < 55	≥ 55	< 45	≥ 45 and < 50	≥ 50 and < 55	≥ 55	< 45	≥ 45 and < 50	≥ 50 and < 55	≥ 55	
OS		24.0	44.6	44.9	59.5	4.0	25.1	22.0	18.4	13	21	22	28	
Gender	Male	45.5	43.8	49.3	58.5	9.1	33.4	22.7	27.4	20	22	24	28	.450
	Female	7.1	45.5	41.1	60.5	0.0	20.0	21.3	10.5	11	21	21	29	<.001
Ca 19.9 (units/mL)	< 90	0.0	61.5	60.3	81.0	0.0	39.1	38.3	10.3	8	3	35	31	<.001
	≥ 90	0.0	9.2	37.2	61.8	0.0	0.0	15.8	32.7	12	18	19	27	.001
Tumor Site	Head	23.8	40.7	46.3	61.1	0.0	22.0	23.1	20.5	13	20	23	28	.001
	Body	0.0	0.0	38.2	66.7	0.0	0.0	20.4	0.0	11	17	23	38	.258
	Tail	50.0	100.0	40.3	40.0	0.0	–	11.9	0.0	–	–	–	–	.406
Type of resection	DCP	30.8	42.2	45.2	61.4	0.0	28.5	25.8	19.3	11	20	23	28	.016
	DP	33.3	75.0	40.5	54.5	33.3	75.0	14.6	0.0	20	NR	22	38	.339
	TP	0.0	25.0	19.4	–	0.0	25.0	0.0	–	NR	NR	NR	NR	<.001
	PPP	20.0	47.7	49.9	–	0.0	15.9	20.6	–	16	22	24	19	.666
Grading	1	–	20.0	66.7	75.0	–	20.0	41.7	45.5	7	21	33	38	<.001
	2	80.0	57.7	45.0	58.1	20.0	42.7	18.4	0.0	30	44	23	28	.313
	3	15.4	40.0	44.5	57.1	–	20.1	21.2	18.7	13	19	21	27	.039
	4	–	50.0	33.6	33.3	–	–	24.0	–	11	10	15	19	.622
Margins status	R0	28.6	51.9	52.9	77.5	14.3	51.9	21.4	25.3	11	NR	25	32	.004
	R1	–	–	24.7	50.0	–	–	12.3	10.7	10	9	20	24	<.001
Tumor diameter	< 30 mm	20.0	25.9	50.0	73.0	–	25.9	0.0	21.3	7	22	23	31	<.001
	≥ 30 mm	50.0	37.5	37.5	58.1	50.0	–	19.4	17.9	11	21	21	28	.493
pT-stage	1	100.0	75.0	66.9	100.0	–	75.0	40.1	0.0	30	NR	35	60	.320
	2	50.0	66.7	53.2	42.9	25.0	35.0	25.5	21.4	11	49	25	19	.639
	3	15.0	38.7	39.6	62.1	0.0	18.5	18.5	20.9	12	17	21	29	<.001
	4	–	0.0	31.6	50.0	–	–	15.8	15.0	NR	20	14	24	.263
pN-stage	N0	25.0	64.9	53.4	83.3	0.0	35.7	29.8	42.3	10	36	27	49	<.001
	N+	23.5	32.4	37.7	53.0	5.9	18.9	15.6	11.4	16	18	20	25	.063
Adjuvant CT	No	31.6	47.7	44.0	28.6	5.3	24.0	22.5	14.3	16	21	21	14	.105
	Yes	–	24.4	47.5	66.7	–	24.4	17.6	20.0	10	17	23	30	<.001

*CT* chemotherapy, *DCP* duodenocephalopancreatectomy, *NR* not reached, *PPP* pylorus preserving pancreatectomy, *RT* radiotherapy, *TP* total pancreatectomy, *DP* distal pancreatectomy

The delivery of higher RT doses resulted as a significant predictor of OS also at multivariate analysis (Table 4). Our multivariable model confirmed a better prognosis in patients treated with doses ≥ 45 Gy. Comparing patients receiving < 45 Gy with those receiving doses ≥ 45 Gy and < 50 Gy (HR: 0.56, 95%CI: 0.34–0.92, *p* = 0.022), or those receiving doses ≥ 50 Gy and < 55 Gy (HR: 0.58, 95%CI: 0.38–0.88, *p* = 0.012), or those receiving doses ≥ 55 Gy (HR: 0.45, 95%CI: 0.28–0.72, *p* < 0.001), a survival improvement was recorded. Furthermore, a higher risk of mortality was observed at multivariate analysis in patients with nodal involvement (HR: 1.56; 95%CI: 1.25–1.95, *p* < 0.001). The other parameters that significantly correlated with OS at univariate analysis did not show a significant correlation at multivariable analysis.

**Table 4:** Multivariable analysis

Variable	HR	CI 95%	*p* Value
pN0	1.00 (Ref.)		
pN1	1.56	1.25–1.95	<.001
Postoperative RT < 45 Gy	1.00 (Ref.)		
Postoperative RT ≥ 45 and < 50 Gy	0.56	0.34–0.92	.022
Postoperative RT ≥ 50 and < 55 Gy	0.58	0.38–0.88	.012
Postoperative RT ≥ 55 Gy	0.45	0.28–0.72	<.001

*RT* radiotherapy

Moreover, considering that patients were treated over a fairly long period of time in which the evolution of imaging techniques could have penalized patients treated in an earlier period, we divided them into 4 groups based on the year of resection: 1995–1998 (54 patients), 1999–2002 (89 patients), 2003–2005 (187 patients), and 2006–2008 (184 patients) and we analysed the correlation between treatment period and administered dose and survival. In the 4 groups, the mean postoperative RT dose underwent a slight but statistically significant increase (50.6 +/− 4.9 Gy, 50.0 +/− 3.2 Gy, 51.2 +/− 3.9 Gy, 52.6 +/− 4.9 Gy, respectively; *p* < 0.001). Furthermore, OS was also significantly improved in patients treated in more recent periods. Indeed, in the 4 groups, median survival was 14, 20, 26, and 24 months, respectively (p: 0.034). However, also this difference was not confirmed at multivariate analysis.

Furthermore, to assess more specifically whether doses higher compared to doses now considered as standard (50 Gy) are more effective, we repeated the univariate and multivariate analysis including only the two subgroups of 50–55 Gy and > 55 Gy. Univariate analysis confirmed the advantage in the cohort receiving > 55 Gy compared to patients treated with 50–55 Gy (2-year OS: 60.0% vs 45.0%, respectively; p: 0.033). Multivariate analysis, considering the group > 55 as a reference, confirmed a trend in terms of higher risk of death in the 50–55 Gy group (HR: 1.31; 95%CI: 0.98–1.74; p: 0.066).

## Discussion

The main result of our study is that increasing RT doses is significantly associated with an improved OS after resection for PDCA with radical intent. This finding has important clinical consequences as well our study clearly shows that postoperative RT dose higher than 45 Gy should be prescribed due to its association with significantly improved prognosis. In addition, our study raises doubts about the dose (50 Gy) recommended by international guidelines [28] especially for patients presenting negative prognostic factors at diagnosis (e.g., high Ca 19–9 level, R1 margin of resection, larger than 3 cm mass). In fact, our results suggest that higher doses (≥ 55 Gy) should be considered when feasible.

We should admit that the comparison in terms of survival, including only the two groups treated with the highest doses (50–55 Gy and > 55 Gy), showed a statistically significant improvement in the second group at univariate analysis but with only a trend at multivariate analysis. However, it should be noted that the possibility of detecting a statistically significant difference was limited by the presence in the first group of 79/336 (23.5%) patients receiving a dose of 54.0–55.0 Gy and in the second group of 6/80 patients (7.5%) receiving a dose < 56 Gy. In other words, more than 30% of the patients included in this sub-analysis received a dose between 54 and 56 Gy, practically equivalent from the clinical point of view.

CRT is a combined modality treatment option for PDAC in the adjuvant setting. The use of postoperative CRT in resected PDAC was initially founded on the results from the GITSG trial which demonstrated an improved survival in patients treated with adjuvant CRT and CT [8]. The results from this trial were confirmed by the non-random enlargement of the sample size with 30 patients in the postoperative CRT arm [9]. That study received some criticism mostly about its small sample size (*n* = 51) and low dose of RT delivered with the obsolete approach of a split-course regimen.

The use of a low dose was justified by the fact that in 1973 when the protocol was designed, the criteria for RT quality assurance were not developed and RT was delivered with supervoltage equipment and anterior-posterior/posterior-anterior fields. Unfortunately, although the technological improvement could have allowed the use of higher doses, in subsequent studies the same RT regimen was used [5, 10].

Few analyses were previously published on the impact of dose in the adjuvant CRT of PDAC. In an analysis of patients with PDAC receiving postoperative CRT with 2 different dose levels, Abrams and colleagues did not observe a significantly different survival between patients undergoing lower dose (50.4 Gy: median survival: 14.4 months) and patients receiving a higher dose (57.6 Gy: median survival: 16.9 months) [26]. However, it should be noted that in their analysis only 23 patients with resected PDAC were included.

More recently, Hall and coworkers analyzed 1385 patients with PDAC treated with postoperative RT +/− CT. Patients receiving a dose of 50–55 Gy showed a significantly higher survival compared to patients receiving doses < 40 Gy, 40 to < 50 Gy, and doses > 55 Gy. They concluded that, on the basis of these results, the optimal dose of adjuvant CRT should range between 50 Gy and 55 Gy [25].

The results of our study differ from those of the Hall’s analysis with regard to patients treated with doses > 55 Gy. In fact, the survival of these patients was significantly improved and worsened in our analysis and in that of Hall and colleagues, respectively. The authors of the cited study hypothesized that patients who underwent higher doses were at least in part those with greater suspicion (for example on CT-simulation) of residual macroscopic disease or that lower survival was due to more serious toxic effects after high-dose RT. The reasons for the opposite result we observed may be due to the following reasons: i) our study involved patients treated in a small number of centers (all academic and research centers with extensive experience in the treatment of PDAC) while the analysis of Hall et al. was performed on data from the National Cancer Data Base and only about one third of the patients had been treated in academic/research cancer programs facilities; ii) all patients included in the Hall’s study were treated from 1998 to 2002, while 72.2% of our patients were treated later when the experience in treating PDAC patients with conformal radiotherapy techniques was probably improved.

Moreover, a significant impact of CRT dose on OS was recorded and confirmed by multivariate analysis. From the analysis we excluded patients who had received a dose < 40 Gy. In fact, we hypothesized that the delivery of doses < 40 Gy was likely due to disease progression in the course of CRT.

This study has obvious limitations, in particular the retrospective nature and the lack of data regarding some parameters (Table 1). Moreover, even if the total number of analyzed patients is 514, only 26 patients received a dose < 45 Gy. This low number may explain the lack of difference in survival in some subgroups of patients (Table 3).

How the different disease (higher T and N stage, higher rate of R1 resection, larger tumors) and treatment (increased use of CT) characteristics in the group treated with > 55 Gy influenced the final result of the analysis is not easy to interpret. However, it should be emphasized that in the multivariate analysis, the lymph node involvement was statistically correlated to survival while the same did not happen for adjuvant CT. Therefore, as a whole, it is not possible to state that this subgroup of patients presented more favourable characteristics compared to others.

This result in some way allows us to better interpret the conflicting results of published studies. In fact, in studies showing an improved survival with the use of adjuvant CRT, doses of 50–50.4 Gy were used [13, 14]. In contrast, the randomized EORTC [10] and ESPAC-1 [5] trials showed lack or a negative impact of CRT with a dose of 40 Gy.

The results of this analysis confirm the ineffectiveness of low doses in improving the clinical outcome and justify the use of higher doses of RT in future studies on adjuvant CRT. The use of higher doses seems feasible as suggested by the acceptable toxicity reported in some studies using doses > 50 Gy [30, 31]. Furthermore, with the use of conformal techniques (3D-conformal or intensity modulated RT), it is possible to administer even higher doses or to intensify the treatment with accelerated regimens. For example, in a dose escalation study based on the 3D-conformal technique with a concomitant boost on the tumor bed, a dose of 55 Gy was reached with a slightly accelerated fractionation (2.2 Gy/fraction) and with concurrent capecitabine. Although this regimen is equivalent to a dose of 57.2 Gy in 2 Gy/fraction (α/β ratio: 3) and despite the administration of two cycles of gemcitabine before CRT, no patient showed grade > 2 toxicity [35]. In addition, it was observed that the use of intensity modulated RT allows a reduction of the radiation dose to healthy organs [36] without an increased incidence of local recurrence [37].

However, higher than standard doses should be prescribed with caution in patients previously treated with neoadjuvant or adjuvant multiple-drug CT, being the impact of intensified systemic treatments on tolerance to subsequent CRT not known.

Finally, based on the inefficiency of low CRT dose in the adjuvant setting, the results of randomized trials should not be further considered as those achievable with modern RT. On the contrary, even in the meta-analysis of Liao and colleagues, data from GITSG, EORTC and ESPAC-1 trials were included [38].

## Conclusion

In a secondary analysis from a retrospective study of patients who underwent radical pancreatectomy, there was a significant relationship between RT dose and OS. This result should lead to reconsider the role and doses of postoperative CRT at least in some categories of patients with higher risk of local recurrence. In addition, based on the current availability of new and more effective systemic therapies, further studies seem justified in order to define the optimal integrated adjuvant therapies and in particular their best sequence.

## Data Availability

The datasets used and/or analysed during the current study are available from the corresponding author on reasonable request.

## Abbreviations

CRT: Chemo-radiotherapy
CT: Chemotherapy
EORTC: European Organization for Research and Treatment of Cancer
ESPAC-1: European Study Group for Pancreatic Cancer-1
GITSG: Gastrointestinal Tumor Study group
LC: Local control
OS: Overall survival
PDAC: Pancreatic ductal adenocarcinoma
pT: Pathological tumor stage
RT: Radiotherapy
